# Remnant cholesterol inflammatory index for predicting pathological complete response to neoadjuvant therapy in breast cancer: A multicenter external validation study

**DOI:** 10.1016/j.breast.2026.104936

**Published:** 2026-09-22

**Authors:** Galip Can Uyar, Kadriye Başkurt, Ahmet Oruç, Nargiz Majidova, Orhun Akdoğan, Enes Yeşilbaş, Kadriye Bir Yücel, Melek Karakurt Eryılmaz, Osman Sütcüoğlu, Ömür Berna Çakmak Öksüzoğlu, Mustafa Altınbaş

**Affiliations:** a Department of Medical Oncology, Etlik City Hospital, Ankara, Turkey; b Department of Medical Oncology, Faculty of Medicine, Necmettin Erbakan University, Konya, Turkey; c Department of Medical Oncology, VM Medical Park Maltepe Hospital, Istanbul, Turkey; d Department of Medical Oncology, Faculty of Medicine, Gazi University, Ankara, Turkey

**Keywords:** Breast cancer, Neoadjuvant therapy, Pathological complete response, Remnant cholesterol inflammatory index, Tumor subtype

## Abstract

**Background:**

Pathological complete response (pCR) after neoadjuvant therapy (NAT) is associated with long-term outcomes in breast cancer (BC). The remnant cholesterol inflammatory index (RCII) integrates lipid-related and inflammatory components, but its role in pCR prediction remains unclear.

**Methods:**

This multicenter retrospective study included women with invasive BC treated with NAT between January 2022 and January 2026. Prespecified multivariable logistic regression models incorporating baseline inflammatory, metabolic, and clinicopathologic variables were developed in a training cohort and evaluated in an independent external validation cohort without recalibration. Model performance was assessed using discrimination and calibration metrics.

**Results:**

Among 422 patients, 157 (37.2%) achieved pCR. In the clinically adjusted model, triple-negative BC (OR 7.56, 95% CI 2.74–20.86), HER2-positive BC (OR 4.13, 95% CI 2.15–7.92), and lower log-RCII (OR 0.48, 95% CI 0.33–0.69) were independently associated with pCR. External validation yielded an AUC of 0.84 (95% CI 0.76–0.91). The calibration slope was 0.94, while the calibration intercept of −0.60 indicated systematic overprediction of pCR probability. Sensitivity and internal validation analyses supported the robustness of the primary findings.

**Conclusion:**

Log-RCII was independently associated with pCR and provided incremental predictive information beyond standard clinicopathologic factors. The model showed favorable discrimination in the external validation cohort, but systematic overprediction indicates that further prospective validation and potential recalibration are warranted before clinical implementation.

## Highlights

• Lower log-transformed RCII independently predicted pathological complete response.
• RCII outperformed C-reactive protein and remnant cholesterol alone.
• External validation showed favorable discrimination.
• Calibration revealed systematic overprediction in the external cohort.
• RCII added predictive information beyond clinicopathologic factors.

## Graphical abstract

**Figure:**
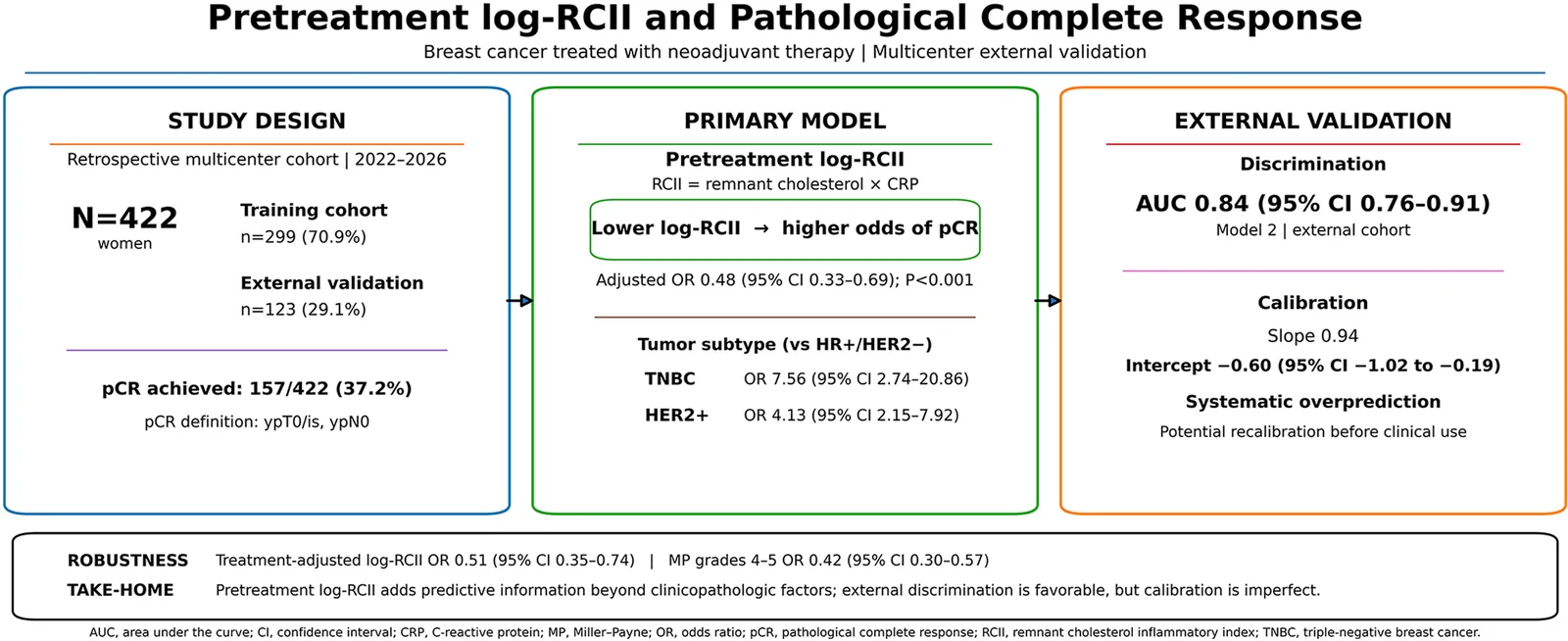

## 1 Introduction

Neoadjuvant systemic therapy (NAT) is a cornerstone of treatment for early and locally advanced breast cancer (BC), enabling tumor downstaging and providing early insight into treatment sensitivity. Pathological complete response (pCR) is associated with improved long-term outcomes, particularly in triple-negative and HER2-positive BC; however, pCR rates vary substantially even within molecular subtypes, highlighting the need for pretreatment predictors of response [[1], [2], [3]].

Several clinical and biological factors have been investigated as predictors of pCR, including tumor subtype, disease stage, histologic grade, and circulating markers of systemic inflammation and nutritional status. C-reactive protein (CRP) has been evaluated as a marker of systemic inflammation and treatment response, while serum albumin reflects both nutritional and inflammatory status [4,5]. Hematologic indices derived from neutrophil, lymphocyte, and platelet counts have also been studied, although their predictive performance has been inconsistent across cohorts and BC subtypes [6,7]. Composite biomarkers such as the nutritional C-reactive protein ratio (NCR) and platelet–albumin–lymphocyte ratio (PALR) integrate inflammatory, immune, and nutritional information and have shown prognostic relevance in oncology [8,9]. Cancer antigen 15-3 (CA15-3) has likewise been associated with tumor burden and treatment outcomes, but its value as a standalone predictor of pCR remains limited [10,11].

Systemic inflammation and altered lipid metabolism are increasingly recognized as interconnected components of cancer biology. Remnant cholesterol (RC), a cholesterol-rich lipoprotein fraction, has been linked to inflammatory signaling and tumor progression [[12], [13], [14]]. The remnant cholesterol inflammatory index (RCII), which combines RC and CRP, has been proposed as a composite marker integrating lipid metabolism and systemic inflammation [15]. Cholesterol-related inflammatory markers have shown prognostic relevance in BC and other oncologic populations [[14], [15], [16]], but the role of RCII in predicting pathological response to NAT remains largely unexplored. Its incremental value alongside established inflammatory–nutritional indices is also uncertain. Moreover, relatively few biomarker-based pCR prediction models have undergone independent multicenter validation with formal assessment of both discrimination and calibration.

Against this background, we aimed to develop and externally validate predictive models for pCR incorporating RCII, established inflammatory–nutritional indices, and clinical variables. We also evaluated model discrimination and calibration in an independent multicenter validation cohort and performed secondary exploratory analyses using the Miller–Payne (MP) grading system.

## 2 Methods

### 2.1 Study design and patient population

This multicenter retrospective cohort study included consecutive patients with BC treated with NAT between January 2022 and January 2026 at tertiary referral centers in Turkey. Patients treated at Etlik City Hospital constituted the training cohort, whereas patients treated at Gazi University Faculty of Medicine, VM Medical Park Maltepe Hospital, and Necmettin Erbakan University Faculty of Medicine comprised the independent multicenter external validation cohort.

During the study period, 587 patients were screened, of whom 422 with available baseline clinical and laboratory data and evaluable pathological response were included in the final analytic cohort (training, n = 299; external validation, n = 123). Exclusion reasons were missing baseline laboratory measurements, incomplete pathological response assessment, and insufficient clinical documentation. The patient selection process is shown in Fig. 1.

**Fig. 1:**
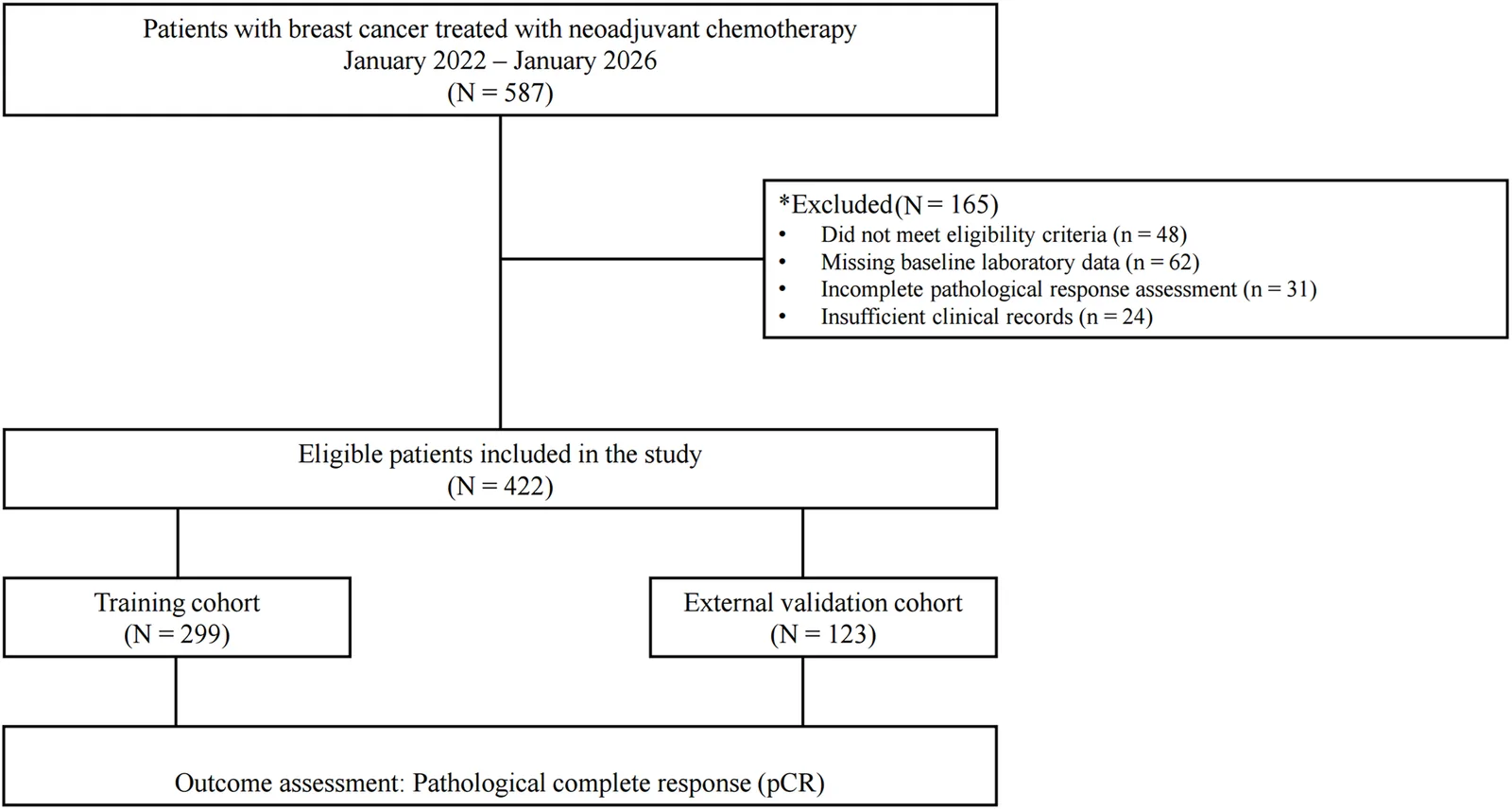
Study flow diagram **Abbreviations:** pCR, pathological complete response. Patients were included consecutively during the study period. Eligibility was assessed prior to initiation of neoadjuvant chemotherapy. Exclusion categories were mutually exclusive. The training cohort was used for model development, and the external validation cohort was used exclusively for independent model validation.

### 2.2 Clinical, pathological, and laboratory data collection

Baseline demographic and clinical data were obtained from electronic health records and archived medical files and included age, menopausal status, body mass index (BMI), Eastern Cooperative Oncology Group (ECOG) performance status, tumor subtype (HR-positive/HER2-negative, triple-negative, or HER2-positive), histologic grade, and clinical T and N stage. All variables were recorded before initiation of NAT.

Pretreatment laboratory measurements included complete blood count components, serum albumin, CRP, CA15-3, and lipid parameters required for RC calculation. Lipid profiles and CRP were obtained from routine morning fasting blood samples. Analyses were performed in institutional laboratories under routine internal and external quality-control procedures. For pooled analyses, results were harmonized to common units: CRP in mg/L, albumin in g/L, and total cholesterol, HDL cholesterol, LDL cholesterol, and RC in mg/dL. Laboratory-specific reference intervals and detection limits were assay- and center-dependent and were not used to categorize biomarker exposure. No central laboratory recalibration or normalization to center-specific reference intervals was performed.

NAT was administered according to tumor subtype and contemporary standard-of-care practice. Anthracycline–taxane regimens predominated, with taxane-only regimens used infrequently. Platinum-containing chemotherapy was used mainly in triple-negative disease and in a small proportion of HER2-positive patients. All patients with HER2-positive disease received trastuzumab, with some receiving dual HER2 blockade with pertuzumab; pembrolizumab was administered to a subset of patients with triple-negative disease. Treatment distributions are provided in Supplementary Table 6.

The primary outcome was pCR, defined as ypT0/is, ypN0, indicating no residual invasive carcinoma in the breast or axillary lymph nodes; residual ductal carcinoma in situ was permitted [1]. Pathological response was determined locally from definitive surgical pathology reports without central review and was coded uniformly across centers using the predefined pCR definition. No study-specific blinding procedure was implemented for pathologists; however, RCII was calculated retrospectively and was not available during routine pathological assessment.

As a secondary exploratory endpoint, Miller–Payne (MP) response was dichotomized as grades 4–5 (favorable) versus grades 1–3 (unfavorable). This threshold distinguished marked or near-complete regression, with grade 4 representing >90% reduction in tumor cellularity and grade 5 the absence of identifiable invasive malignant cells at the tumor site [17].

### 2.3 Calculation of inflammatory and metabolic indices

Inflammatory and metabolic indices were calculated using pretreatment values. NCR was calculated as (BMI [kg/m^2^] × serum albumin [g/L])/CRP [mg/L] [8]. PALR was calculated as (platelet count [×10^9^/L] × serum albumin [g/L])/absolute lymphocyte count [×10^9^/L] [9].

RC was calculated as total cholesterol minus high-density lipoprotein cholesterol and low-density lipoprotein cholesterol (mg/dL). RCII was calculated as the product of RC and CRP. Given its right-skewed distribution, RCII was natural log–transformed (log-RCII) and used in all regression and modeling analyses [15]. To improve interpretability in regression models and forest plots, NCR and PALR were scaled per 100-unit increase.

### 2.4 Statistical analysis

All statistical analyses were performed using R version 4.5.2 (R Foundation for Statistical Computing, Vienna, Austria). The pROC, PRROC, mice, rms, boot, dcurves, PredictABEL, and car packages were used, as appropriate, for ROC and DeLong analyses, precision–recall analyses, multiple imputation, regression modeling and calibration, bootstrap validation, decision-curve analysis, reclassification analyses, and multicollinearity assessment, respectively. A two-sided P value < 0.05 was considered statistically significant. Continuous variables were summarized as medians with interquartile ranges (IQRs), and categorical variables as counts and percentages. Group comparisons were performed using the Mann–Whitney *U* test and chi-square or Fisher's exact test, as appropriate.

Univariable logistic regression was performed in the training cohort to evaluate candidate predictors of pCR. Two prespecified multivariable logistic regression models were developed based on clinical relevance and prior evidence. Model 1 included NCR, PALR, log-RCII, tumor subtype, and age. Model 2 additionally included ECOG performance status, clinical T and N stage, and histologic grade. The chemotherapy backbone was not included in Model 2 because anthracycline–taxane-based therapy predominated and showed limited variability. Multicollinearity was assessed using variance inflation factors, with all VIFs <5. Training-cohort coefficients were applied directly to the external validation cohort without recalibration.

Discrimination was assessed using ROC AUC and PR-AUC, with 95% CIs for AUC estimated by DeLong's method. Predictive accuracy was assessed using the Brier score. Calibration was evaluated in the external validation cohort using calibration plots, calibration slope, calibration intercept, and mean absolute calibration error, with 200 bootstrap resamples. Internal validation used bootstrap optimism correction with 1000 resamples.

Multiple imputation by chained equations was performed as a sensitivity analysis in the training cohort. The imputation model included all Model 2 predictors and the observed pCR outcome; pCR itself was not imputed. Missingness was limited to one age value. Twenty imputed datasets were generated using predictive mean matching, and estimates were pooled using Rubin's rules. Patients excluded because of unavailable pathological response or insufficient clinical documentation were not reintroduced, and the external validation cohort was not used for imputation.

Treatment heterogeneity was explored by additionally adjusting Model 2 for platinum exposure, dual HER2 blockade, and pembrolizumab exposure, each coded as yes/no. Formal treatment-by-subtype interactions and subtype-specific treatment-adjusted models were not estimated because several exposure combinations were structurally empty or sparse. The treatment-adjusted model included 298 evaluable patients, 122 pCR events, and 13 predictor parameters excluding the intercept.

Incremental predictive value was assessed by comparing clinicopathologic models incorporating CRP, RC, or log-RCII using DeLong's test. Continuous NRI and IDI were used to evaluate incremental performance beyond clinicopathologic variables. DCA assessed net clinical benefit, and RCS with three knots evaluated potential nonlinearity. Interaction terms assessed whether the association between log-RCII and pCR differed by tumor subtype.

In secondary exploratory analyses, discrimination was assessed within tumor subtypes in the external validation cohort; subtype-specific calibration was not estimated because of limited event and non-event counts. Model performance was also evaluated for the secondary MP endpoint, defined as grades 4–5 versus grades 1–3, using ROC and precision–recall analyses.

### 2.5 Ethics statement

The study was conducted in accordance with the Declaration of Helsinki and was approved by the Ankara Etlik City Hospital Scientific Research Evaluation Committee (approval number: AEŞH-BADEK-2024-380). Institutional authorization for retrospective data contribution was obtained at each participating center in accordance with local requirements. Only de-identified patient-level clinical and laboratory data were transferred to the coordinating center for pooled analysis, and no directly identifiable information was shared between institutions. Given the retrospective design and use of de-identified routinely collected clinical data, the requirement for written informed consent was waived in accordance with applicable institutional and ethics procedures at the participating centers.

## 3 Results

### 3.1 Study population and patient characteristics

A total of 587 female patients with BC treated with NAT were screened for eligibility. After exclusion of patients with missing baseline laboratory measurements, incomplete pathological response assessment, or insufficient clinical documentation, 422 patients were included in the final analytic cohort (Fig. 1). Of these, 299 (70.9%) constituted the training cohort and 123 (29.1%) the independent external validation cohort.

Baseline characteristics are presented in Table 1. The cohorts were similar in age (median, 53 years [IQR, 43–62] vs 51 years [IQR, 42–60]; P = 0.424), and no significant differences were observed in menopausal status, tumor subtype, histologic grade, clinical stage, or baseline inflammatory and metabolic indices (all P > 0.05).

**Table 1:** Baseline characteristics of the training and external validation cohorts and pCR distribution.

Variable (n = 422)	Training cohort (n = 299) (70.9%)	External cohort (n = 123) (29.1%)	P value
**Age, median (IQR), years**	53 (43–62)	51 (42–60)	0.424
**Menopausal status, n (%)**			0.892
Premenopausal	141 (47.2)	59 (48.0)	
Postmenopausal	158 (52.8)	64 (52.0)	
**ECOG performance status, n (%)**			0.411
0	267 (89.3)	113 (91.9)	
≥1	32 (10.7)	10 (8.1)	
**Tumor subtype, n (%)**			0.986
HR-positive/HER2-negative	146 (48.8)	61 (49.6)	
Triple-negative	42 (14.0)	16 (13.0)	
HER2-positive	111 (37.1)	46 (37.4)	
**Histologic grade, n (%)**			0.382
Grade 1–2	138 (46.2)	51 (41.5)	
Grade 3	161 (53.8)	72 (58.5)	
**Clinical T stage, n (%)**			0.299
cT1–2	198 (66.2)	88 (71.5)	
cT3–4	101 (33.8)	35 (28.5)	
**Clinical N stage, n (%)**			0.645
cN0	77 (25.8)	29 (23.6)	
cN+	222 (74.2)	94 (76.4)	
**Baseline CA15-3 (U/mL), median (IQR)**	21 (12–27)	22 (14–39)	0.318
**Baseline BMI, median (IQR), kg/m^2^**	26.0 (24.0–28.0)	25.5 (24.0–27.5)	0.310
**Baseline albumin, median (IQR), g/L**	44 (42–46)	44 (42–45)	0.525
**Baseline CRP, median (IQR), mg/L**	3.0 (2.0–9.0)	3.0 (2.0–8.0)	0.610
**NCR, median (IQR)**	379.9 (152.4–746.3)	398.5 (77.4–794.0)	0.477
**PALR, median (IQR)**	5868 (4409–7610)	6310 (4623–8501)	0.199
**log-RCII, median (IQR)**	2.24 (1.37–3.20)	2.44 (1.38–3.80)	0.218
**pCR, n (%)**	**123 (41.1)**	**34 (27.6)**	**0.008**

**Abbreviations:** BMI: body mass index, CA15-3: cancer antigen 15-3, CRP: C-reactive protein, ECOG: Eastern Cooperative Oncology Group, HER2: human epidermal growth factor receptor 2, IQR: interquartile range, NCR: nutritional C-reactive protein ratio, PALR: platelet–albumin–lymphocyte ratio, pCR: pathological complete response, RCII: remnant cholesterol inflammatory index.

Continuous variables are presented as medians with interquartile ranges (IQRs), and categorical variables as counts with percentages. Continuous variables were compared using the Mann–Whitney *U* test, and categorical variables using the chi-square test or Fisher's exact test, as appropriate. The nutritional C-reactive protein ratio (NCR) was calculated as (body mass index × serum albumin) divided by C-reactive protein, and the platelet–albumin–lymphocyte ratio (PALR) as (platelet count × serum albumin) divided by the absolute lymphocyte count. RCII denotes the remnant cholesterol inflammatory index, and log-RCII represents logarithmically transformed RCII values. Pathological complete response (pCR) is reported for descriptive purposes only and was not considered a baseline characteristic for cohort comparability.

### 3.2 Baseline characteristics according to pathological complete response status

Overall, 157 patients (37.2%) achieved pCR. Characteristics according to pCR status are presented in Table 2. Patients with pCR were younger than those without pCR (median, 49.1 vs 54.1 years). HR-positive/HER2-negative disease accounted for 26.1% of patients with pCR versus 62.6% of those without pCR, whereas HER2-positive and triple-negative disease were more frequent among patients achieving pCR. Median log-RCII was lower in patients with pCR (1.67 [IQR, 1.08–2.35]) than in those without pCR (2.65 [IQR, 1.81–4.08]).

**Table 2:** Baseline demographic, clinical, and laboratory characteristics according to pathological complete response (pCR) status.

Variable	Overall (N = 422)	pCR Absent (n = 265, 62.8%)	pCR Present (n = 157, 37.2%)
**Age, years, median (IQR)**	52.2 (42.7–62.8)	54.1 (43.6–63.4)	49.1 (41.7–56.2)
**Menopausal status, n (%)**			
Premenopausal	222 (52.6)	119 (44.9)	103 (65.6)
Postmenopausal	200 (47.4)	146 (55.1)	54 (34.4)
**ECOG performance status, n (%)**			
0	380 (90.0)	236 (89.1)	144 (91.7)
≥1	42 (10.0)	29 (10.9)	13 (8.3)
**Breast cancer subtype, n (%)**			
HR-positive/HER2-negative	207 (49.1)	166 (62.6)	41 (26.1)
HER2-positive	157 (37.2)	74 (27.9)	83 (52.9)
Triple-negative	58 (13.7)	25 (9.4)	33 (21.0)
**Histological grade, n (%)**			
Grade 1–2	189 (44.8)	143 (54.0)	46 (29.3)
Grade 3	233 (55.2)	122 (46.0)	111 (70.7)
**Clinical T stage, n (%)**			
T1–2	286 (67.8)	167 (63.0)	119 (75.8)
T3–4	136 (32.2)	98 (37.0)	38 (24.2)
**Clinical N stage, n (%)**			
N0	105 (24.9)	61 (23.0)	44 (28.0)
N+	317 (75.1)	204 (77.0)	113 (72.0)
**Clinical stage, n (%)**			
Stage II	205 (48.6)	119 (44.9)	86 (54.8)
Stage III	217 (51.4)	146 (55.1)	71 (45.2)
**BMI, kg/m^2^, median (IQR)**	25.8 (24.0–28.7)	28.0 (26.5–30.6)	23.1 (21.1–26.2)
**Albumin, g/L, median (IQR)**	44 (42–46)	44 (41–46)	44 (42–46)
**CRP, mg/L, median (IQR)**	3 (2–8)	4 (2–13)	2 (1–6)
**NCR, median (IQR)**	379.9 (125.6–756.1)	295.3 (66.4–580.2)	544.1 (228.6–868.9)
**PALR, median (IQR)**	6043.8 (4453.6–7871.0)	5868.2 (4200.2–7792.1)	6259.1 (5008.9–8053.6)
**log-RCII, median (IQR)**	2.29 (1.37–3.41)	2.65 (1.81–4.08)	1.67 (1.08–2.35)
**Baseline CA15-3 (U/mL), median (IQR)**	21 (12–27)	22 (14–39)	20 (11–28)

**Abbreviations:** BMI: body mass index, CA15-3: cancer antigen 15-3, CI: confidence interval, CRP: C-reactive protein, ECOG: Eastern Cooperative Oncology Group, HER2: human epidermal growth factor receptor 2, IQR: interquartile range, NCR: nutritional C-reactive protein ratio, OR: odds ratio, PALR: platelet–albumin–lymphocyte ratio, pCR: pathological complete response, RCII: remnant cholesterol inflammatory index.

Continuous variables are presented as median (interquartile range [IQR]) and categorical variables as counts (percentages). pCR indicates pathological complete response. NCR (nutritional C-reactive protein ratio) was calculated as (body mass index × serum albumin)/C-reactive protein. PALR denotes platelet–albumin–lymphocyte ratio, and RCII denotes remnant cholesterol inflammatory index. log-RCII represents logarithmically transformed RCII values.

### 3.3 Predictors of pathological complete response

Univariable analyses in the training and overall cohorts are provided in Supplementary Tables 1 and 2

In multivariable analyses, tumor subtype and log-RCII were significant in both prespecified models (Supplementary Table 3, Fig. 2). In Model 1, ORs for pCR were 7.42 (95% CI, 2.97–18.55) for triple-negative disease, 5.46 (95% CI, 2.85–10.47) for HER2-positive disease, and 0.47 (95% CI, 0.34–0.65) per unit increase in log-RCII (all P < 0.001).

**Fig. 2:**
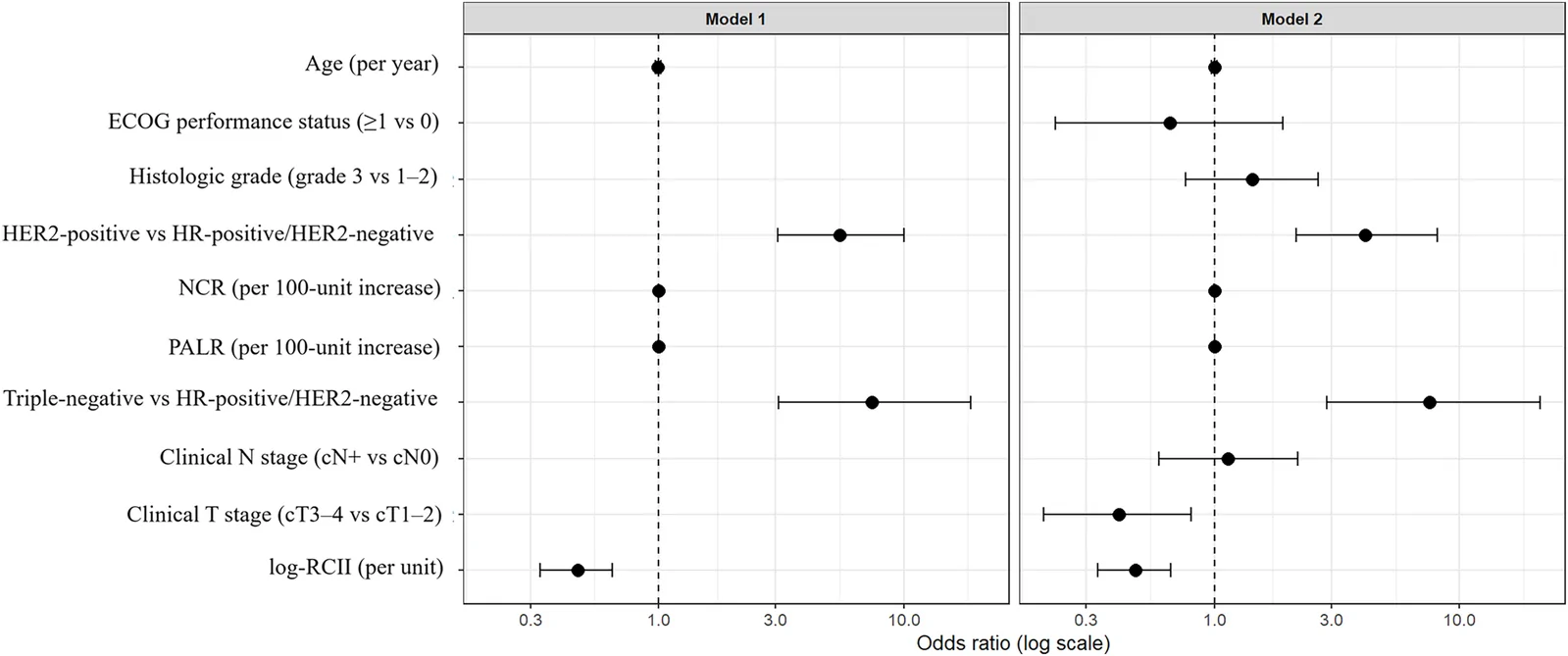
Multivariable logistic regression models for prediction of pathological complete response (pCR) **Abbreviations:** CI, confidence interval; ECOG, Eastern Cooperative Oncology Group; NCR, nutritional C-reactive protein ratio; OR, odds ratio; PALR, platelet–albumin–lymphocyte ratio; pCR, pathological complete response; RCII, remnant cholesterol inflammatory index Forest plot showing adjusted odds ratios (ORs) with 95% confidence intervals (CIs) for Model 1 and Model 2 derived from multivariable logistic regression analyses for prediction of pathological complete response (pCR) in the training cohort. Model 1 included age, tumor subtype, nutritional C-reactive protein ratio (NCR), platelet–albumin–lymphocyte ratio (PALR), and log-transformed remnant cholesterol inflammatory index (log-RCII). Model 2 included all variables in Model 1 and was additionally adjusted for ECOG performance status, clinical T stage, clinical N stage, and histologic grade. Odds ratios are reported per predefined scaling.

In Model 2, corresponding ORs were 7.56 (95% CI, 2.74–20.86; P < 0.001), 4.13 (95% CI, 2.15–7.92; P < 0.001), and 0.48 (95% CI, 0.33–0.69; P < 0.001), respectively. Advanced clinical T stage was also independently associated with lower odds of pCR (OR 0.41, 95% CI, 0.21–0.79; P = 0.011).

### 3.4 Model performance and calibration

Model performance is summarized in Table 3. In the training cohort, AUCs were 0.79 (95% CI, 0.74–0.84) for Model 1 and 0.81 (95% CI, 0.76–0.86) for Model 2, with PR-AUCs of 0.63 and 0.65, respectively. In the external validation cohort, AUCs were 0.82 (95% CI, 0.74–0.90) and 0.84 (95% CI, 0.76–0.91), with PR-AUCs of 0.66 for both models (Table 3, Fig. 3).

**Table 3:** Discrimination and calibration performance of Model 1 and Model 2 in the training and external validation cohorts Discrimination and calibration metrics for Model 1 and Model 2.

Metric	Model 1 – Training	Model 1 – External	Model 2 – Training	Model 2 – External
AUC (95% CI)	0.79 (0.74–0.84)	0.82 (0.74–0.90)	0.81 (0.76–0.86)	0.84 (0.76–0.91)
PR-AUC	0.63	0.66	0.65	0.66
Brier score	0.17	0.16	0.16	0.15
Calibration slope	—	—	—	0.94
Calibration Intercept (95% CI)	—	−0.52 (−0.95 – 0.01)	—	−0.60 (−1.02 –-0.19)
Mean absolute calibration error	—	—	—	0.037

Abbreviations: AUC, area under the receiver operating characteristic curve; CI, confidence interval; MAE, mean absolute calibration error; PR-AUC, area under the precision–recall curve.

**Model definitions:** Model 1 included nutritional C-reactive protein ratio (NCR), platelet–albumin–lymphocyte ratio (PALR), log-transformed remnant cholesterol inflammatory index (log-RCII), tumor subtype, and age. Model 2 included all variables in Model 1 and was additionally adjusted for ECOG performance status, clinical T stage, clinical N stage, and histologic grade.

Model performance was evaluated using discrimination and calibration metrics. Discrimination was assessed using the area under the receiver operating characteristic curve (AUC) with 95% confidence intervals and the precision–recall AUC (PR-AUC). Calibration was assessed exclusively in the external validation cohort using calibration plots and quantitative metrics, including the calibration slope and mean absolute calibration error (MAE), estimated by bootstrap resampling with 200 repetitions. Calibration slope was derived by regressing observed outcomes on predicted probabilities, with values closer to 1 indicating better calibration. Lower MAE values indicate better agreement between predicted and observed probabilities. Dashes (−) indicate metrics not applicable to the training cohort. Calibration intercept (calibration-in-the-large) evaluates systematic over- or underestimation of the predicted probabilities, with an ideal value of 0.

**Fig. 3:**
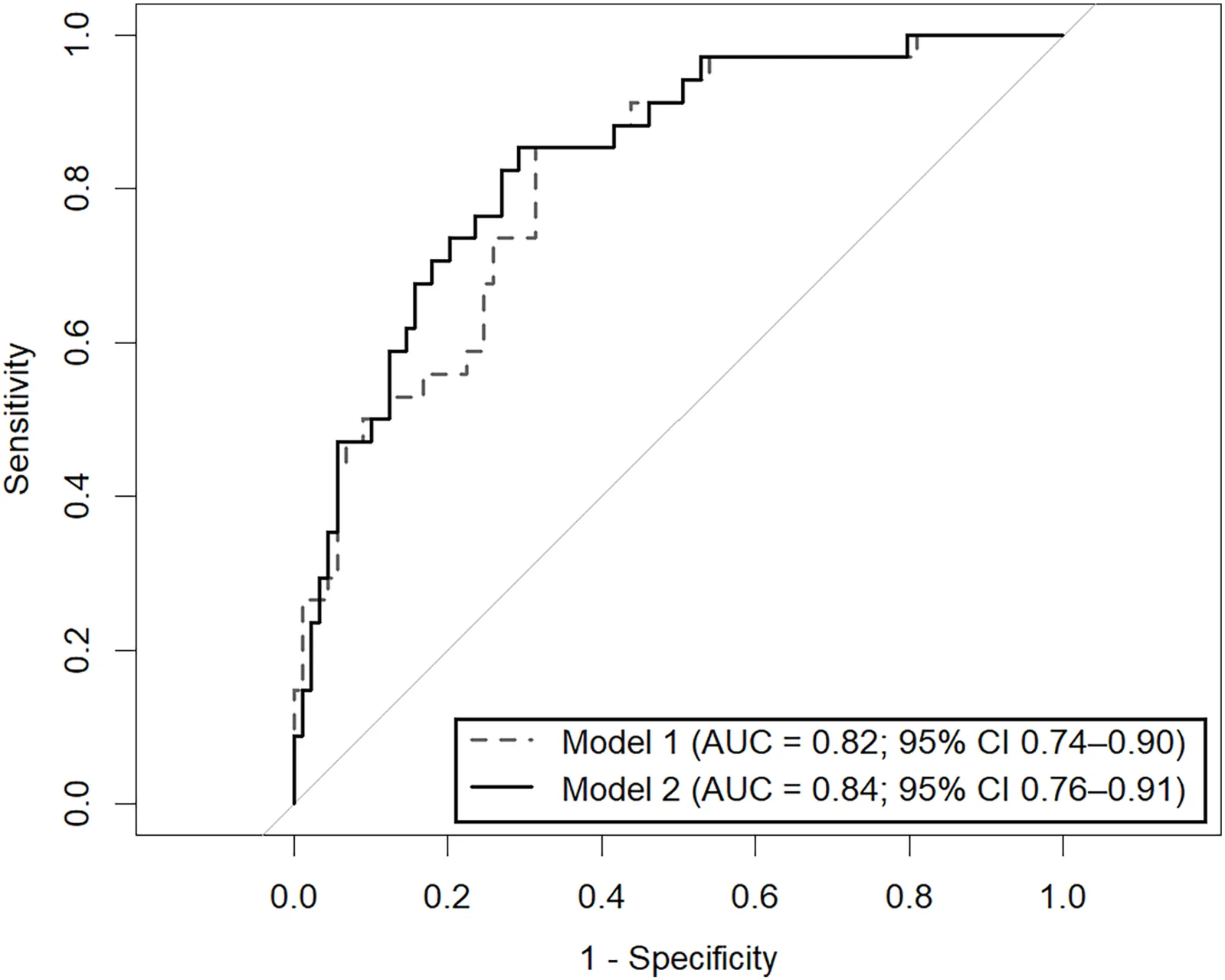
Discrimination performance of prediction models for pathological complete response (pCR) in the external validation cohort **Abbreviations:** AUC, area under the receiver operating characteristic curve; CI, confidence interval; pCR, pathological complete response; ROC, receiver operating characteristic. Receiver operating characteristic (ROC) curves showing the discriminative performance of Model 1 and Model 2 for prediction of pathological complete response (pCR) in the training and external validation cohorts. Model performance was quantified using the area under the ROC curve (AUC) with 95% confidence intervals.

The baseline clinicopathologic model yielded an AUC of 0.738. AUCs increased to 0.786 with CRP, 0.783 with RC, and 0.810 with log-RCII. The log-RCII model showed higher discrimination than the CRP (P = 0.010) and RC (P = 0.046) models by DeLong testing (Supplementary Fig. 1). Addition of log-RCII also yielded a continuous NRI of 0.667 and an IDI of 0.108.

DCA showed higher net benefit for the model incorporating log-RCII across threshold probabilities of approximately 0.10–0.75 (Supplementary Fig. 2). RCS analysis showed no evidence of nonlinearity (P for nonlinearity = 0.245; Supplementary Fig. 3).

No statistically significant interactions were observed between log-RCII and tumor subtype (P = 0.722 and P = 0.971); however, these findings should not be interpreted as evidence of homogeneous associations across subtypes. In the external cohort, Model 2 AUCs were 0.82 (95% CI, 0.69–0.94) in HR-positive/HER2-negative disease (n = 61; 13 pCR events), 0.77 (95% CI, 0.51–0.98) in triple-negative disease (n = 16; 10 events), and 0.89 (95% CI, 0.76–0.99) in HER2-positive disease (n = 46; 11 events) (Supplementary Table 4).

In the external validation cohort, Model 2 demonstrated a calibration slope of 0.94, calibration intercept of −0.60 (95% CI, −1.02 to −0.19), and mean absolute calibration error of 0.037, indicating systematic overprediction of pCR probability. The calibration intercept for Model 1 was −0.52 (95% CI, −0.95 to 0.01) (Table 3). Calibration and precision–recall plots are shown in Supplementary Fig. 4. Bootstrap optimism correction showed limited optimism, with the apparent AUC of Model 2 decreasing from 0.81 to 0.79 and the Brier score increasing from 0.16 to 0.17.

### 3.5 Sensitivity analyses

Multiple-imputation results were consistent with complete-case analyses. The pooled OR for log-RCII was 0.48 (95% CI, 0.34–0.67), compared with 0.48 (95% CI, 0.33–0.69) in the complete-case analysis. Corresponding pooled ORs were 7.86 (95% CI, 2.91–21.26) for triple-negative disease, 4.10 (95% CI, 2.13–7.92) for HER2-positive disease, and 0.40 (95% CI, 0.20–0.80) for cT3–4 disease (Supplementary Table 5).

Treatment characteristics are presented in Supplementary Table 6. Exposure was subtype-specific, with dual HER2 blockade restricted to HER2-positive disease, pembrolizumab to triple-negative disease, and platinum used predominantly in triple-negative disease. The treatment-adjusted model included 298 evaluable patients, 122 pCR events, and 13 predictor parameters. After additional adjustment for these exposures, log-RCII remained associated with pCR (OR 0.51, 95% CI, 0.35–0.74; P < 0.001), with similar external validation performance (Supplementary Table 7).

### 3.6 Favorable pathological response according to the Miller–Payne grading system

Favorable pathological response was defined as MP grades 4–5 versus grades 1–3. Detailed analyses and subtype-specific distributions are presented in Supplementary Tables 8 and 9 In multivariable analysis, log-RCII, NCR, and tumor subtype were significant. ORs were 5.23 (95% CI, 1.81–17.90; P = 0.004) for triple-negative disease and 3.70 (95% CI, 1.93–7.29; P < 0.001) for HER2-positive disease versus HR-positive/HER2-negative disease. Lower log-RCII (OR 0.42, 95% CI, 0.30–0.57; P < 0.001) and NCR (OR 0.89, 95% CI, 0.81–0.97; P = 0.013) were also associated with favorable response (Supplementary Table 8, Supplementary Fig. 5). Model discrimination is shown in Supplementary Fig. 6.

## 4 Discussion

In this multicenter study with external validation, we developed and evaluated two prespecified multivariable models for predicting pCR after NAT. Log-RCII was independently associated with pCR in both the inflammation-based and clinically adjusted models. Tumor subtype was also independently associated with pCR, with higher response rates in HER2-positive and triple-negative BC than in HR-positive/HER2-negative disease. Clinical T stage remained independently associated with pCR in the clinically adjusted model. These findings suggest that log-RCII may provide complementary information beyond established clinicopathologic factors for pretreatment assessment of pathological response.

The biological plausibility of log-RCII as a predictor of neoadjuvant response may reflect the interplay between lipid metabolism and systemic inflammation. RC is a cholesterol-rich lipoprotein fraction with pro-inflammatory properties that may promote macrophage activation, cytokine release, and tumor-promoting immune dysregulation [12,13,18]. When combined with CRP, log-RCII integrates lipid-related metabolic disturbance with systemic inflammatory activity, potentially capturing complementary aspects of host–tumor biology [19]. Altered cholesterol homeostasis may influence cancer cell proliferation, membrane signaling, and immune modulation, while systemic inflammation may further shape the tumor microenvironment and treatment sensitivity [4,20,21]. Consistent with this biological rationale, log-RCII remained independently associated with pCR across the prespecified models and also with favorable response according to the secondary MP-based endpoint. These findings support further evaluation of log-RCII as an integrated metabolic–inflammatory marker of neoadjuvant treatment response.

Our findings should be considered within the broader literature evaluating pretreatment inflammatory and nutritional biomarkers for pCR prediction. Although several circulating indices have been associated with response, their performance has been inconsistent across cohorts and molecular subtypes, particularly after adjustment for tumor biology and disease burden [[22], [23], [24], [25], [26], [27], [28]]. Externally validated clinicopathologic and machine-learning models have generally reported validation AUCs of approximately 0.75–0.85 [26,27], placing the external AUC of 0.84 observed for Model 2 within the upper range of previously reported performance. Importantly, independent validation and formal calibration assessment remain relatively uncommon. In our study, the prespecified model retained favorable discrimination in an independent cohort without refitting, while calibration analysis identified systematic overprediction. These findings highlight both the potential value of integrating log-RCII with established clinicopathologic factors and the need for further external validation and potential recalibration before clinical implementation [29,30].

Beyond log-RCII, several clinicopathologic factors showed associations with pCR that were consistent with established neoadjuvant patterns. Triple-negative and HER2-positive tumors had higher response rates, whereas more advanced clinical T stage was associated with lower pCR, in keeping with known differences in chemosensitivity and tumor burden [1,2,31]. Higher histologic grade also showed an association with greater response, consistent with the increased treatment sensitivity of more proliferative tumors [32,33]. In contrast, ECOG performance status and baseline CA15-3 did not provide independent predictive information for pCR [[34], [35], [36], [37]]. Similarly, NCR and PALR did not retain independent significance after adjustment for tumor biology and overlapping inflammatory variables, consistent with the heterogeneous and context-dependent performance reported for these indices [22,23,38].

This study has several notable strengths. Its multicenter design and independent validation cohort enabled evaluation of model performance in a separate patient population without recalibration, with both discrimination and calibration explicitly assessed. The use of complementary performance metrics and formal calibration assessment strengthens the methodological evaluation of the models. In addition, the MP-based secondary endpoint provided a complementary measure of pathological response, supporting the consistency of the observed association with log-RCII across related response endpoints.

Several limitations should be acknowledged. The retrospective design introduces the potential for selection bias and unmeasured confounding. Although neoadjuvant treatment was administered according to contemporary standard-of-care practice and tumor subtype, treatment heterogeneity may have influenced response patterns. The anthracycline–taxane backbone was used in the large majority of patients but was not included as a covariate in the primary models, and residual confounding related to regimen selection, treatment intensity, or sequencing cannot be excluded. Exposure to contemporary treatment components, including platinum-containing regimens, dual HER2 blockade, and pembrolizumab, was lower in the external validation cohort and may have contributed to differences in pCR rates. The absence of detailed molecular or genomic profiling further limited treatment-specific modeling. Biomarkers were assessed at a single pretreatment time point, precluding evaluation of dynamic changes during therapy. Although laboratory values were harmonized to common reporting units, assays were performed locally rather than in a central laboratory, and residual interlaboratory variability cannot be excluded. Pathological response was also assessed locally without central pathology review, which may have introduced interinstitutional variability despite use of a uniform pCR definition. Although the validation cohort was geographically independent and derived from separate tertiary referral centers, all participating institutions were located within the same national healthcare system. Subtype-specific analyses were exploratory and limited by small subgroup sizes and event counts; therefore, the absence of statistically significant interaction should not be interpreted as evidence of homogeneous log-RCII associations across subtypes. Finally, despite limited optimism on bootstrap validation and favorable discrimination in the external cohort, the negative calibration intercept indicated systematic overprediction of pCR probability, suggesting that recalibration may be required in populations with different baseline response rates or treatment patterns. Further prospective validation in larger and more diverse populations is required before clinical implementation.

## 5 Conclusion

In this multicenter study with external validation, log-RCII remained independently associated with pCR after adjustment for tumor subtype and key clinical variables, and the models demonstrated favorable discrimination in the validation cohort. Its association with favorable pathological response according to the MP-based secondary endpoint further supported the consistency of this finding. In contrast, NCR and PALR showed limited independent contribution after multivariable adjustment. These findings support further investigation of log-RCII as an integrated metabolic–inflammatory marker of neoadjuvant treatment response. However, systematic overprediction in the external validation cohort indicates that further prospective validation and potential recalibration are required before clinical implementation.

## CRediT authorship contribution statement

**Galip Can Uyar:** Writing – review & editing, Writing – original draft, Visualization, Project administration, Methodology, Investigation, Formal analysis, Data curation, Conceptualization. **Kadriye Başkurt:** Writing – review & editing, Investigation, Data curation. **Ahmet Oruç:** Writing – review & editing, Investigation, Data curation. **Nargiz Majidova:** Writing – review & editing, Investigation, Data curation. **Orhun Akdoğan:** Writing – review & editing, Investigation, Data curation. **Enes Yeşilbaş:** Writing – review & editing, Investigation, Data curation. **Kadriye Bir Yücel:** Writing – review & editing, Supervision. **Melek Karakurt Eryılmaz:** Writing – review & editing, Supervision. **Osman Sütcüoğlu:** Writing – review & editing, Supervision. **Ömür Berna Çakmak Öksüzoğlu:** Writing – review & editing, Supervision. **Mustafa Altınbaş:** Writing – review & editing, Supervision.

## Availability of data and materials

The data that supports the findings of this study are available from the corresponding author upon reasonable request, subject to institutional and ethical restrictions.

## Ethics approval and consent to participate

The study was conducted in accordance with the Declaration of Helsinki and was approved by the Ankara Etlik City Hospital Scientific Research Evaluation Committee (approval number: AEŞH-BADEK-2024-380). Institutional authorization for retrospective data contribution was obtained at each participating center in accordance with local institutional requirements. Only de-identified patient-level clinical and laboratory data were transferred to the coordinating center for pooled analysis, and no directly identifiable information was shared between institutions. Given the retrospective design of the study and the use of de-identified routinely collected clinical data, the requirement for written informed consent was waived in accordance with the applicable institutional and ethics procedures at the participating centers.

## Consent for publication

Not applicable.

## Trial registration

Not applicable.

## Impact statement

This study demonstrates that cholesterol-related inflammatory features, captured by the remnant cholesterol inflammatory index, can be integrated into prediction models to estimate pathological response to neoadjuvant therapy in breast cancer. Validation in an independent multicenter cohort suggests potential utility for combining inflammatory and lipid-related biomarkers in risk stratification and supports further investigation of cholesterol–inflammation interactions in treatment response.

## Funding

This research received no specific grant from any funding agency in the public, commercial, or not-for-profit sectors.

## Declaration of competing interest

The authors declare that they have no known competing financial interests or personal relationships that could have appeared to influence the work reported in this paper.
