# Supplementary material for: Remnant cholesterol inflammatory index for predicting pathological complete response to neoadjuvant therapy in breast cancer: A multicenter external validation study
Source: Breast. 2026 Sep 22;90:104936. doi: 10.1016/j.breast.2026.104936 (PMC13633918; doi:10.1016/j.breast.2026.104936)
Supplement: Supplementary file 1 [file mmc1.docx]

**Supplementary Table 1. Univariable logistic regression analysis for predictors of pathological complete response (pCR) in the training cohort**

| **Variable** | **OR (95% CI)** | **P value** |
| --- | --- | --- |
| Age (per year) | 0.98 (0.96–1.00) | 0.041 |
| Postmenopausal vs premenopausal | 0.41 (0.25–0.66) | <0.001 |
| ECOG performance status |  |  |
| ≥1 vs 0 | 1.03 (0.43–2.39) | 0.946 |
| Tumor subtype |  | <0.001 |
| TNBC vs HR+/HER2- | 5.10 (2.45–10.63) | <0.001 |
| HER2+ vs HR+/HER2- | 7.78 (4.41–13.72) | <0.001 |
| Grade 3 vs grade 1–2 | 2.44 (1.51–4.01) | <0.001 |
| cT3–4 vs cT1–2 | 0.40 (0.24–0.66) | <0.001 |
| cN+ vs cN0 | 0.69 (0.41–1.15) | 0.158 |
| Baseline BMI (per 5 kg/m²) | 1.07 (0.86–1.34) | 0.544 |
| Baseline albumin (per g/L) | 1.08 (1.01–1.16) | 0.021 |
| Baseline CRP (per mg/L) | 0.95 (0.91–0.98) | 0.009 |
| Baseline CA15-3 (per 10 U/mL) | 0.94 (0.81–1.10) | 0.466 |
| NCR (per 100-units increase) | 1.04 (1.02–1.05) | 0.002 |
| PALR (per 100-units increase) | 1.00 (0.98–1.02) | 0.076 |
| log-RCII (per unit) | 0.57 (0.46–0.69) | <0.001 |

**Abbreviations:** BMI, body mass index; CA15-3, cancer antigen 15-3; CI, confidence interval; CRP, C-reactive protein; ECOG, Eastern Cooperative Oncology Group; HER2, human epidermal growth factor receptor 2; NCR, nutritional C-reactive protein ratio; OR, odds ratio; PALR, platelet–albumin–lymphocyte ratio; pCR, pathological complete response; RCII, remnant cholesterol inflammatory index.

Univariable logistic regression analyses were performed exclusively in the training cohort. Odds ratios (ORs) with 95% confidence intervals (CIs) are reported. Continuous variables were analyzed using predefined scaling to improve interpretability: age per 1-year increase; body mass index (BMI) per 5 kg/m² increase; serum albumin per 1 g/L increase; C-reactive protein (CRP) per 1 mg/L increase; nutritional C-reactive protein ratio (NCR), defined as (BMI × serum albumin)/CRP, and platelet–albumin–lymphocyte ratio (PALR) per 100-unit increase; cancer antigen 15-3 (CA15-3) per 10 U/mL increase; and remnant cholesterol inflammatory index after logarithmic transformation (log-RCII) per 1-unit increase. A two-sided P value <0.05 was considered statistically significant.

**Supplementary Table 2. Univariable logistic regression analysis for pathological complete response (pCR) in the overall cohort**

| **Variable** | **OR (95% CI)** | **P value** |
| --- | --- | --- |
| Age (per year) | 0.98 (0.97–0.99) | 0.005 |
| Postmenopausal (vs premenopausal) | 0.31 (0.20–0.49) | <0.001 |
| ECOG ≥1 (vs 0) | 0.75 (0.38–1.48) | 0.475 |
| **Subtype** |  |  |
| HR-positive/HER2-negative | Reference | — |
| HER2-positive | 4.57 (2.85–7.33) | <0.001 |
| Triple-negative | 5.33 (2.88–9.87) | <0.001 |
| Grade 3 (vs 1–2) | 2.82 (1.88–4.22) | <0.001 |
| cT3–4 (vs cT1–2) | 0.55 (0.35–0.86) | 0.009 |
| cN+ (vs cN0) | 0.79 (0.50–1.25) | 0.212 |
| Stage III (vs II) | 0.67 (0.43–1.04) | 0.071 |
| BMI (per 5 kg/m²) | 0.86 (0.61–1.34) | 0.337 |
| Albumin (per g/L) | 0.98 (0.86–1.18) | 0.243 |
| CRP (per mg/L) | 0.92 (0.89–0.95) | <0.001 |
| NCR (per 100 units) | 1.02 (1.01–1.03) | <0.001 |
| PALR (per 100 units) | 1.02 (1.01–1.04) | 0.033 |
| log-RCII (per unit) | 0.47 (0.37–0.60) | <0.001 |
| Baseline CA15-3 (per 10 U/mL) | 0.93 (0.79–1.10) | 0.406 |

**Abbreviations:** BMI, body mass index; CA15-3, cancer antigen 15-3; CI, confidence interval; CRP, C-reactive protein; NCR, nutritional C-reactive protein ratio; OR, odds ratio; PALR, platelet–albumin–lymphocyte ratio; pCR, pathological complete response; RCII, remnant cholesterol inflammatory index.

Odds ratios (ORs) were estimated using univariable logistic regression. Continuous variables were analyzed using predefined scaling to improve interpretability: age per 1-year increase; body mass index (BMI) per 5 kg/m² increase; serum albumin per 1 g/L increase; C-reactive protein (CRP) per 1 mg/L increase; nutritional C-reactive protein ratio (NCR), defined as (BMI × albumin)/CRP, and platelet–albumin–lymphocyte ratio (PALR) per 100-unit increase; cancer antigen 15-3 (CA15-3) per 10 U/mL increase; and remnant cholesterol inflammatory index after logarithmic transformation (log-RCII) per 1-unit increase. A two-sided P value <0.05 was considered statistically significant.

**Supplementary Table 3. Multivariable logistic regression analysis for predictors of pathological complete response (pCR) in the training cohort**

**Model 1: Inflammation-based model**

| **Variable** | **Adjusted OR (95% CI)** | **P value** |
| --- | --- | --- |
| Age (per year) | 1.00 (0.98–1.03) | 0.707 |
| **Tumor subtype** |  |  |
| **Triple-negative vs HR-positive/HER2-negative** | **7.42 (2.97–18.55)** | **<0.001** |
| **HER2-positive vs HR-positive/HER2-negative** | **5.46 (2.85–10.47)** | **<0.001** |
| NCR (per 100-unit increase) | 1.000 (0.998–1.001) | 0.208 |
| PALR (per 100-unit increase) | 1.00 (0.99–1.01) | 0.087 |
| **log-RCII (per unit)** | **0.47 (0.34–0.65)** | **<0.001** |

**Model 2: Clinically adjusted model**

| **Variable** | | **Adjusted OR (95% CI)** | **P value** |
| --- | --- | --- | --- |
| Age (per year) | 1.00 (0.97–1.03) | | 0.958 |
| Tumor subtype |  | |  |
| **Triple-negative vs HR-positive/HER2-negative** | **7.56 (2.74–20.86)** | | **<0.001** |
| **HER2-positive vs HR-positive/HER2-negative** | **4.13 (2.15–7.92)** | | **<0.001** |
| NCR (per 100-unit increase) | 0.95 (0.87–1.04) | | 0.303 |
| PALR (per 100-unit increase) | 1.009 (0.999–1.020) | | 0.065 |
| **log-RCII (per unit)** | **0.48 (0.33–0.69)** | | **<0.001** |
| ECOG performance status (≥1 vs 0) | 0.66 (0.23–1.89) | | 0.441 |
| **Clinical T stage (cT3–4 vs cT1–2)** | **0.41 (0.21–0.79)** | | **0.011** |
| Clinical N stage (cN+ vs cN0) | 1.14 (0.59–2.19) | | 0.702 |
| Histologic grade (grade 3 vs 1–2) | 1.43 (0.78–2.63) | | 0.265 |

**Abbreviations:** BMI, body mass index; CA15-3, cancer antigen 15-3; CI, confidence interval; CRP, C-reactive protein; ECOG, Eastern Cooperative Oncology Group; HER2, human epidermal growth factor receptor 2; NCR, nutritional C-reactive protein ratio; OR, odds ratio; PALR, platelet–albumin–lymphocyte ratio; pCR, pathological complete response; RCII, remnant cholesterol inflammatory index

Multivariable logistic regression analyses were performed in the training cohort to identify independent predictors of pathological complete response (pCR). Model 1 included inflammatory and nutritional indices and tumor subtype, while Model 2 was additionally adjusted for clinical and pathological variables. Continuous variables were analyzed using predefined scaling to improve interpretability: age per 1-year increase; nutritional C-reactive protein ratio (NCR), defined as (body mass index × serum albumin)/C-reactive protein, and platelet–albumin–lymphocyte ratio (PALR) per 100-unit increase; and remnant cholesterol inflammatory index after logarithmic transformation (log-RCII) per 1-unit increase. Odds ratios (ORs) with 95% confidence intervals (CIs) are reported. A two-sided P value <0.05 was considered statistically significant.

**Supplementary Table 4. External validation performance of Model 2 stratified by tumor subtype**

| **Tumor subtype** | **N (%)** | **pCR events, n (%)** | **AUC (95% CI)** | **PR-AUC** |
| --- | --- | --- | --- | --- |
| HR-positive/HER2-negative | 61 (49.6) | 13 (21.3) | 0.82 (0.69–0.94) | 0.55 |
| Triple-negative | 16 (13.0) | 10 (62.5) | 0.77 (0.51–0.98) | 0.82 |
| HER2-positive | 46 (37.4) | 11 (23.9) | 0.89 (0.76–0.99) | 0.72 |

**Abbreviations:** AUC: area under the receiver operating characteristic curve, CI: confidence interval, HER2: human epidermal growth factor receptor 2, pCR: pathological complete response, PR-AUC: area under the precision–recall curve

Model discrimination was evaluated in the external validation cohort using the area under the receiver operating characteristic curve (AUC), with 95% confidence intervals estimated using DeLong’s method, and the precision–recall AUC (PR-AUC). Values for N are presented as counts with column percentages, and pCR events as counts with row percentages within each tumor subtype. Subtype-specific analyses were exploratory and should be interpreted cautiously because of the limited sample size and number of pCR events within individual subgroups, particularly the triple-negative subgroup.

**Supplementary Table 5. Comparison of complete-case and multiple-imputation analyses for the clinically adjusted model**

| **Variable** | **Complete-case adjusted OR (95% CI)** | **Multiple-imputation pooled OR (95% CI)** | **MI P value** |
| --- | --- | --- | --- |
| Age, per year | 1.00 (0.97–1.03) | 1.00 (0.97–1.03) | 0.958 |
| Triple-negative vs HR+/HER2− | 7.56 (2.74–20.86) | **7.86 (2.91–21.26)** | <0.001 |
| HER2+ vs HR+/HER2− | 4.13 (2.15–7.92) | **4.10 (2.13–7.92)** | <0.001 |
| NCR, per 100-unit increase | 0.95 (0.87–1.04) | **0.95 (0.87–1.04)** | 0.291 |
| PALR, per 100-unit increase | 1.009 (0.999–1.020) | **1.009 (0.999–1.020)** | 0.064 |
| log-RCII, per 1-unit increase | 0.48 (0.33–0.69) | **0.48 (0.34–0.67)** | <0.001 |
| ECOG ≥1 vs 0 | 0.66 (0.23–1.89) | **0.66 (0.23–1.90)** | 0.440 |
| cT3–4 vs cT1–2 | 0.41 (0.21–0.79) | **0.40 (0.20–0.80)** | 0.009 |
| cN+ vs cN0 | 1.14 (0.59–2.19) | **1.12 (0.59–2.14)** | 0.732 |
| Grade 3 vs grade 1–2 | 1.43 (0.78–2.63) | **1.43 (0.77–2.66)** | 0.263 |

Abbreviations: CI, confidence interval; ECOG, Eastern Cooperative Oncology Group; HER2, human epidermal growth factor receptor 2; MI, multiple imputation; NCR, nutritional C-reactive protein ratio; OR, odds ratio; PALR, platelet–albumin–lymphocyte ratio; pCR, pathological complete response; RCII, remnant cholesterol inflammatory index; TNBC, triple-negative breast cancer. Multiple imputation by chained equations was performed in the training cohort using 20 imputed datasets. The pCR outcome was not imputed. The prespecified clinically adjusted model was fitted in each imputed dataset, and estimates were combined using Rubin’s rules.

**Supplementary Table 6. Neoadjuvant treatment characteristics according to breast cancer subtype and study cohort**

| **Variable** | **HR+/HER2− Training (n=146)** | **HR+/HER2− External (n=61)** | **HER2+ Training (n=111)** | **HER2+ External (n=46)** | **TNBC Training (n=42)** | **TNBC External (n=16)** |
| --- | --- | --- | --- | --- | --- | --- |
| Anthracycline–taxane backbone | 131 (89.7) | 55 (90.2) | 102 (91.9) | 42 (91.3) | 42 (100) | 15 (93.8) |
| Taxane-only regimen | 15 (10.3) | 6 (9.8) | 0 | 0 | 0 | 1 (6.2) |
| Platinum-containing regimen | 0 | 0 | 7 (6.3) | 2 (4.3) | 30 (71.4) | 7 (43.8) |
| Trastuzumab exposure | — | — | 111 (100) | 46 (100) | — | — |
| Pertuzumab exposure | — | — | 72 (64.9) | 21 (45.7) | — | — |
| Dual HER2 blockade | — | — | 72 (64.9) | 21 (45.7) | — | — |
| Pembrolizumab exposure | — | — | — | — | 14 (33.3) | 2 (12.5) |

**Abbreviations:** HER2, human epidermal growth factor receptor 2; TNBC, triple-negative breast cancer.

Values are presented as n (%). Anthracycline–taxane backbone refers to anthracycline- and taxane-based neoadjuvant chemotherapy. Platinum-containing regimen refers to any neoadjuvant chemotherapy regimen incorporating carboplatin or cisplatin. Trastuzumab exposure and pertuzumab exposure were recorded among patients with HER2-positive disease. Dual HER2 blockade was defined as concomitant administration of trastuzumab and pertuzumab during neoadjuvant therapy. Pembrolizumab exposure was assessed among patients with triple-negative breast cancer. Percentages were calculated using the corresponding subtype-specific cohort as the denominator. In the treatment-adjusted sensitivity analysis, platinum exposure, dual HER2 blockade, and pembrolizumab exposure were coded as binary variables (yes/no). Dashes indicate treatment–subtype combinations that were not applicable because the treatment was restricted to the corresponding molecular subtype.

**Supplementary Table 7. Exploratory treatment-adjusted sensitivity analysis incorporating platinum exposure, dual HER2 blockade, and pembrolizumab exposure**

| **Variable** | **Adjusted OR (95% CI)** | **P value** |
| --- | --- | --- |
| **log-RCII (per 1-unit increase)** | **0.51 (0.35–0.74)** | **<0.001** |
| **Triple-negative vs HR-positive/HER2-negative** | **6.84 (2.39–19.54)** | **<0.001** |
| **HER2-positive vs HR-positive/HER2-negative** | **3.72 (1.88–7.36)** | **0.002** |
| **Clinical T stage (cT3–4 vs cT1–2)** | **0.43 (0.22–0.84)** | **0.014** |
| Platinum exposure | 1.42 (0.63–3.18) | 0.397 |
| Dual HER2 blockade | 1.67 (0.82–3.41) | 0.157 |
| Pembrolizumab exposure | 1.51 (0.47–4.88) | 0.491 |

| **Model performance in the external validation cohort** | | |
| --- | --- | --- |
| **Metric** | **Original Model 2** | **Treatment-adjusted sensitivity model** |
| External AUC (95% CI) | 0.84 (0.76–0.91) | 0.83 (0.75–0.91) |
| External PR-AUC | 0.66 | 0.67 |
| External Brier score | 0.15 | 0.15 |
| External Calibration slope | 0.94 | 0.91 |

**Abbreviations:** AUC, area under the receiver operating characteristic curve; CI, confidence interval; HER2, human epidermal growth factor receptor 2; OR, odds ratio; pCR, pathological complete response; PR-AUC, area under the precision–recall curve; RCII, remnant cholesterol inflammatory index.

Treatment-adjusted sensitivity analysis was performed by additionally incorporating platinum exposure, dual HER2 blockade, and pembrolizumab exposure, each coded as a binary variable (yes/no), into the clinically adjusted multivariable logistic regression model. Because treatment exposure was strongly subtype-specific and several treatment-by-subtype combinations were structurally empty or sparse, formal treatment-by-subtype interaction terms were not estimated because they would yield non-identifiable or unstable parameter estimates. Accordingly, this analysis was intended to assess whether the association between log-RCII and pCR was materially altered after accounting for major treatment exposures and should not be interpreted as an analysis of treatment effects or treatment-by-subtype effect modification. Odds ratios (ORs) with 95% confidence intervals (CIs) are reported. Model discrimination was assessed using the area under the receiver operating characteristic curve (AUC) and precision–recall AUC (PR-AUC), overall predictive accuracy using the Brier score, and calibration using the calibration slope. The treatment-adjusted model was fitted in 298 evaluable patients in the training cohort, including 122 pCR events, and contained 13 predictor parameters excluding the intercept.

**Supplementary Table 8. Univariable and multivariable logistic regression analyses for predictors of favorable pathological response according to the Miller–Payne grading system in the training cohort**

| **Variable** | **Univariable OR (95% CI)** | **P value** | **Multivariable OR (95% CI)** | **P value** |
| --- | --- | --- | --- | --- |
| log-RCII (per unit increase) | **0.57 (0.46–0.69)** | **<0.001** | **0.42 (0.30–0.57)** | **<0.001** |
| NCR (per 100-unit increase) | **0.94 (0.89–0.99)** | **0.021** | **0.89 (0.81–0.97)** | **0.013** |
| PALR (per 100-unit increase) | 1.01 (1.00–1.02) | 0.076 | 1.00 (0.99–1.01) | 0.556 |
| **Tumor subtype** |  |  |  |  |
| Triple-negative vs HR-positive/HER2-negative | **7.17 (3.02–16.1)** | **<0.001** | **5.23 (1.81–17.9)** | **0.004** |
| HER2-positive vs HR-positive/HER2-negative | **4.41 (2.50–8.52)** | **<0.001** | **3.70 (1.93–7.29)** | **<0.001** |
| Age (per year) | 0.99 (0.97–1.01) | 0.415 | — | — |
| ECOG performance status ≥1 vs 0 | 0.81 (0.46–1.43) | 0.478 | — | — |
| Baseline CA15-3 (per 10 U/mL increase) | 0.96 (0.86–1.08) | 0.525 | — | — |
| Clinical T stage (cT3–4 vs cT1–2) | 0.62 (0.40–0.96) | 0.032 | 1.07 (0.57–2.02) | 0.844 |
| Clinical N stage (cN+ vs cN0) | 0.84 (0.54–1.31) | 0.440 | — | — |
| Histologic grade (grade 3 vs 1–2) | 2.11 (1.37–3.25) | <0.001 | 1.63 (0.90–2.95) | 0.105 |

**Abbreviations:** CA15-3, cancer antigen 15-3; CI, confidence interval; ECOG, Eastern Cooperative Oncology Group; HER2, human epidermal growth factor receptor 2; MP, Miller–Payne; NCR, nutritional C-reactive protein ratio; OR, odds ratio; PALR, platelet–albumin–lymphocyte ratio; RCII, remnant cholesterol inflammatory index.

Univariable and multivariable logistic regression analyses were performed in the training cohort to identify predictors of the secondary binary endpoint of favorable pathological response. Favorable pathological response was defined as MP grades 4–5, whereas grades 1–3 were classified as unfavorable response. This dichotomization was used to distinguish marked or near-complete pathological regression from lesser degrees of response, as described in the Methods. Odds ratios (ORs) with 95% confidence intervals (CIs) are reported. NCR and PALR were modeled per 100-unit increase, and log-RCII per 1-unit increase. Variables included in the multivariable model were selected a priori based on clinical relevance. A two-sided P value <0.05 was considered statistically significant.

**Supplementary Table 9. Distribution of Miller–Payne pathological response by tumor subtype in the training and external validation cohorts**

**Training cohort (n = 298)**

| **Tumor subtype** | **MP grades 1–3 (Unfavorable), n (%)** | **MP grades 4–5 (Favorable), n (%)** | **Total** |
| --- | --- | --- | --- |
| HR-positive/HER2-negative | 86 (58.9) | 60 (41.1) | 146 |
| Triple-negative | 7 (16.7) | 35 (83.3) | 42 |
| HER2-positive | 27 (24.5) | 83 (75.5) | 110 |
| **Overall** | **120 (40.3)** | **178 (59.7)** | **298** |

**External validation cohort (n = 123)**

| **Tumor subtype** | **MP grades 1–3 (Unfavorable), n (%)** | **MP grades 4–5 (Favorable), n (%)** | **Total** |
| --- | --- | --- | --- |
| HR-positive/HER2-negative | 34 (55.7) | 27 (44.3) | 61 |
| Triple-negative | 5 (31.3) | 11 (68.8) | 16 |
| HER2-positive | 20 (43.5) | 26 (56.5) | 46 |
| **Overall** | **59 (48.0)** | **64 (52.0)** | **123** |

**Abbreviations:** HER2, human epidermal growth factor receptor 2; MP, Miller–Payne; TNBC, triple-negative breast cancer.
Miller–Payne pathological response was analyzed as a secondary binary endpoint and dichotomized as MP grades 4–5 (favorable pathological response) versus grades 1–3 (unfavorable pathological response). Miller–Payne grading was evaluable for 298 of 299 patients in the training cohort; one patient without a valid Miller–Payne grade was excluded from MP-based analyses. Values are presented as counts with row percentages within each tumor subtype.

**Supplementary Figure 1. Receiver operating characteristic curve comparison of C-reactive protein-, remnant cholesterol-, and remnant cholesterol inflammatory index–based models for prediction of pathological complete response**

**Abbreviations:** AUC, area under the receiver operating characteristic curve; CRP, C-reactive protein; RC, remnant cholesterol; RCII, remnant cholesterol inflammatory index.

Receiver operating characteristic curves are shown for clinicopathologic models incorporating age, clinical T stage, clinical N stage, histologic grade, and tumor subtype, with the addition of CRP, RC, or log-RCII. Areas under the curve were compared using the DeLong test. DeLong test P values were 0.010 for the comparison between the log-RCII and CRP models and 0.046 for the comparison between the log-RCII and RC models.

**Supplementary Figure 2. Decision-curve analysis comparing the baseline clinicopathologic model and the clinicopathologic model incorporating log-RCII for prediction of pathological complete response**

**Abbreviations:** DCA, decision-curve analysis; RCII, remnant cholesterol inflammatory index.

Net benefit is plotted against threshold probability for the baseline clinicopathologic model, the clinicopathologic model incorporating log-RCII, and the treat-all and treat-none strategies.

**Supplementary Figure 3. Restricted cubic spline analysis evaluating the association between log-RCII and the probability of pathological complete response**

**Abbreviations:** CI, confidence interval; RCII, remnant cholesterol inflammatory index.

The solid line represents the estimated probability of pathological complete response across the range of log-RCII values, and the shaded area represents the 95% confidence interval. Restricted cubic spline analysis was performed using three knots. The P value for nonlinearity was 0.245.

**Supplementary Figure 4. Calibration and precision–recall analyses of prediction models for pathological complete response (pCR)**

**Abbreviations:** MAE, mean absolute calibration error; pCR, pathological complete response; PR-AUC, area under the precision–recall curve.

(A) Calibration plot of Model 2 in the external validation cohort, showing the relationship between predicted and observed probabilities of pathological complete response. Model 2 demonstrated a calibration slope of 0.94, a calibration intercept of −0.60 (95% CI, −1.02 to −0.19), and a mean absolute calibration error of 0.037. The negative calibration intercept indicates systematic overprediction of pCR probability in the external validation cohort. (B) Precision–recall curves for Model 1 and Model 2 in the external validation cohort. The PR-AUC was 0.66 for both models.

**Supplementary Figure 5. Multivariable logistic regression analysis for favorable pathological response according to the Miller–Payne grading system in the training cohort**

**Abbreviations:** CI, confidence interval; MP, Miller–Payne; OR, odds ratio.

Adjusted odds ratios (ORs) were obtained from a multivariable logistic regression model evaluating predictors of favorable pathological response, defined as Miller–Payne grades 4–5 versus grades 1–3, in the training cohort. Continuous variables were modeled using predefined scaling, and covariates were selected a priori based on clinical relevance.

**Supplementary Figure 6. Receiver operating characteristic and precision–recall analyses for favorable pathological response according to the Miller–Payne grading system in the training cohort**

**Abbreviations:** AUC, area under the receiver operating characteristic curve; MP, Miller–Payne; PR-AUC, area under the precision–recall curve.

**(A)** Receiver operating characteristic (ROC) curve illustrating the discriminative performance of the multivariable prediction model for favorable pathological response, defined as Miller–Payne grade 4–5 versus grades 1–3, in the training cohort. **(B)** Precision–recall (PR) curve evaluating the performance of the same model for predicting favorable pathological response in the training cohort.

Discriminative performance for favorable pathological response was assessed using receiver operating characteristic (ROC) and precision–recall (PR) curves. Favorable pathological response was defined as Miller–Payne grade 4–5 versus grades 1–3. Analyses were performed exclusively in the training cohort.
